# Recent Trends in Enzyme Immobilization—Concepts for Expanding the Biocatalysis Toolbox

**DOI:** 10.3390/molecules26092822

**Published:** 2021-05-10

**Authors:** Hans-Jürgen Federsel, Thomas S. Moody, Steve J.C. Taylor

**Affiliations:** 1 RISE Research Institutes of Sweden, Department of Chemical Process and Pharmaceutical Development, P.O. Box 5607, S-114 86 Stockholm, Sweden; 2 Almac Sciences Ltd., 20 Seagoe Industrial Estate, Craigavon BT63 5QD, UK; sjctaylor2@gmail.com; 3 Arran Chemical Company Limited, Unit 1 Monksland Industrial Estate, N37 DN24 Athlone, Ireland

**Keywords:** enzyme, biocatalysis, immobilization, flow processes, support technologies, 3D-printing

## Abstract

Enzymes have been exploited by humans for thousands of years in brewing and baking, but it is only recently that biocatalysis has become a mainstream technology for synthesis. Today, enzymes are used extensively in the manufacturing of pharmaceuticals, food, fine chemicals, flavors, fragrances and other products. Enzyme immobilization technology has also developed in parallel as a means of increasing enzyme performance and reducing process costs. The aim of this review is to present and discuss some of the more recent promising technical developments in enzyme immobilization, including the supports used, methods of fabrication, and their application in synthesis. The review highlights new support technologies such as the use of well-established polysaccharides in novel ways, the use of magnetic particles, DNA, renewable materials and hybrid organic–inorganic supports. The review also addresses how immobilization is being integrated into developing biocatalytic technology, for example in flow biocatalysis, the use of 3D printing and multi-enzymatic cascade reactions.

## 1. Introduction

Despite the many millions of organic compounds already known, either of natural origin or artificially prepared by humans, there is still a need to design and synthesize novel molecules. They could represent hitherto completely unknown structural architectures and functional features or be mere analogs of existing chemical entities carrying minor or major modifications. The task of making compounds previously not known is, without a doubt, driven by the curiosity to explore whether a specific molecule can be made. This is, however, not the end of the story, as there will always be a desire to investigate the properties of the products isolated, for example, whether they are pharmacologically active, can be used for agricultural purposes or are of interest as new materials. It is against this backdrop that a synthetic chemistry toolbox with enhanced capabilities is required to be able to successfully tackle the many challenges on the way to new chemical structures. Enzymes, the catalysts of nature, are such an enabling tool, which can offer unique possibilities when focusing on the synthesis of target molecules.

Biocatalysis was once the domain of specialists working with limited in-house collections of enzymes and cultures. A revolution in molecular biology has enabled the rapid development of much larger and more diverse enzyme collections and has also enabled astonishing improvements in enzyme process performance to be realized. This has elevated biocatalysis from assuming a niche position to be a mainstream technology widely applied, relevant to a diverse range of chemical transformations [1,2,3,4]. In addition, enzyme immobilization has been used to pave the way forward for further improvements.

The primary motivation for enzyme immobilization has always been about increasing enzyme performance. This might be realized by increased activity, stereoselectivity or stability, with the enzyme in a suitable physical form that enables it to be recovered at the end of biotransformation and reused. This translates to reduced cost of the final product by reducing the cost contribution of the biocatalyst. A useful immobilized enzyme allows for fewer fermentations to be performed or for easier product isolation or higher product quality to be achieved in fewer processing steps.

Enzyme immobilization, as a specialism within the field of biocatalysis, is growing in prominence, partly driven by the rapidly increasing acceptance and application of biocatalysis itself [5]. This is apparent across several sectors, for example in pharmaceuticals, chemicals, biofuels, food, flavors, fragrances and cosmetics. The purpose of this short review is not to reiterate the many approaches and examples of successfully immobilized enzymes but rather to appraise the recent literature to highlight some of the latest thinking and directions in the field of enzyme immobilization.

Enzyme immobilization has been reviewed recently by several authors, and these provide a good starting point for evaluation of recent developments [6,7,8,9]. Broadly speaking, two main themes can be identified. The first is the development of novel supports, and this may include solid supports that have not hitherto been used for enzyme immobilization or existing supports that have been applied in new ways. The second theme concerns the integration of enzyme immobilization with rapidly moving state-of-the-art biocatalytic technologies. This includes topics such as protein engineering, flow biocatalysis and multiple cascade reactions where sequential biotransformations emulate biosynthetic pathways.

## 2. Discussion: Novel Support Technologies

### 2.1. Polysaccharides

The use of polysaccharides as supports for enzyme immobilization has been reviewed [7]. Various polysaccharides are widely available and suitably low cost and as a result have been used for many years as supports. The most prominent materials are alginates, chitosan, cellulose, agarose, guar gum, agar, carrageenan, gelatin, dextran, xanthan and pectins. The types of sugars in these polymers and their bonding characteristics give rise to a broad range of immobilization chemistries. 

**Figure 1:**
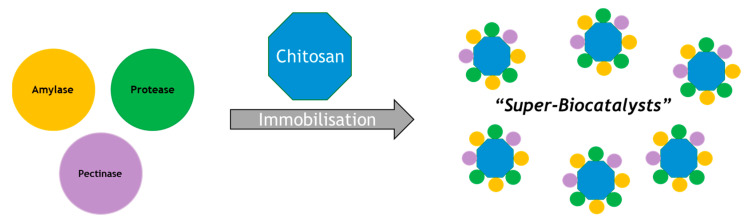
Immobilization studies using three enzymes to make “Super-Biocatalysts”.

The use of a single polysaccharide support to immobilize multiple enzymes was investigated [10]. In this study, sodium alginate and glutaraldehyde-activated chitosan were used to assess their potential for immobilizing three enzymes at once, namely an α-amylase, protease and pectinase, as highlighted in Figure 1. This is a useful example that highlights the potential for developing new “super-biocatalysts”, where a single material performs multiple biocatalytic functions. This might be, for example, to debitter, de-haze and reduce protein levels of a liquid in a single operation. Alternatively, multiple enzymes on a single support may be used to perform a sequence of reactions as discussed below. We may anticipate further such examples in the future where several enzymes are loaded onto a single support [11,12,13,14,15,16,17,18,19,20,21].

### 2.2. DNA

**Figure 2:**
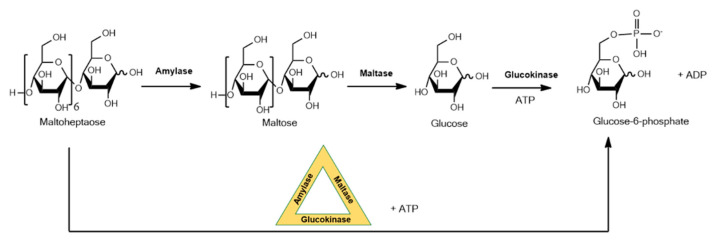
Three-enzyme sequential cascade using immobilized amylase, maltase and glucokinase on a self-assembled DNA origami triangle.

DNA is emerging as a promising substrate for enzyme immobilization. This form of immobilization exploits DNA that forms nanostructures that will enable formation of multiple different enzyme–DNA conjugates with a high level of spatial precision. Close spatial proximity of enzymes allows phenomena such as substrate channeling to occur where the product of an enzyme reaction is efficiently delivered to the active site of the next enzyme in a sequence, a strategy that is widespread in nature, for example, in complex natural product biosynthetic pathways. This approach with DNA is aimed at diagnostic applications, and in a recent example a three-enzyme sequential cascade was realized by site-specifically immobilizing DNA-conjugated amylase, maltase and glucokinase on a self-assembled DNA origami triangle, forming a phosphorylated sugar from an oligosaccharide as shown in Figure 2 [22]. The kinetics of seven different enzyme configurations were evaluated experimentally and compared to simulations of optimized activity. A 30-fold increase in the pathway’s kinetic activity was observed for enzymes assembled to the DNA.

Whilst this method for enzyme immobilization is in its infancy, it is likely that more examples will be developed in the coming years with promise towards improving other more elaborate multi-enzymatic cascades and could potentially allow for the custom synthesis of complex (bio)molecules that cannot be realized with conventional organic chemistry approaches.

### 2.3. Chitosan

Chitosan is a well-known support for enzyme immobilization, and its application was recently reviewed [23,24]. This biopolymer, either derived from waste crustacean shells or mushrooms and other fungi, shows unique physicochemical properties. It is an attractive support due to its low-cost, large-scale availability, biodegradability, non-toxicity and bio-adhesive properties. It entraps bioactive biomolecules such as protein and nucleic acid through various mechanisms such as chemical cross-linking, ionic cross-linking and ionic complexation. 

Most applications utilize particulate forms of chitosan. Newer approaches, however, include methods such as electrospinning, a powerful approach to obtain thin-layered chitosan mats, with good mechanical stability. The advantages of such mats include a high surface-to-volume ratio, ability to tailor high porosity and high mass transfer. Chitosan mats were crosslinked with glutaraldehyde to form mechanically stable mats, which were further functionalized with glucose oxidase [25]. The aim was to obtain fibrous mats exhibiting antimicrobial properties by virtue of their ability to generate hydrogen peroxide. Such mats that produce hydrogen peroxide in clinically relevant concentrations are of interest as a dressing material for wound-care applications. This technology that features biocatalytic mats could be useful in a range of scenarios that require this type of support format.

### 2.4. Renewables

Renewable materials such as agricultural and food wastes have several characteristics that make them of potential interest in enzyme immobilization technology. Aside from being widely available and low in cost, they may possess high porosity with high surface area and the presence of different chemical groups (amino, hydroxyl, carboxyl, thiol and phosphate groups). This endows them with the ability to participate in the main enzyme immobilization approaches, including surface adsorption, ion exchange, complexation−chelation and microprecipitation. Materials may be organic or inorganic. Typical examples of the former that have come under scrutiny in this broad class of materials include coconut fiber, corn cobs, corn stover, rice husk and spent coffee grounds. These are generally lignocellulosic with varying composition containing lignin, cellulose and hemicellulose, imparting differing physical properties to each. Inorganic materials include eggshells and ashes with carbon chars in between. The use of these materials has recently been reviewed [26], and examples are described below. Applications are varied and include biofuel production, chemical synthesis, waste treatment and food production.

Coconut fiber can be obtained from unripe coconut. It is available in high abundance with a global annual production of about 350,000 metric tons. It is a material that is underutilized, with 90% going to waste landfill. It has physical and chemical properties of great potential in various applications and is seeing application in enzyme immobilization including lipases [27], laccases [28] and amylase [29]. 

Rice husk ash was used as a support for the immobilization of a recombinant *Rhizopus oryzae* (rROL) lipase for biodiesel production using alperujo oil as substrate feed [30]. The support was compared to the commercial hydrophobic support OD403 (RelOD). Although the specific activity was around one-half lower in rice husk compared to RelOD, the enzyme was used as a biocatalyst in biodiesel reactions. It was shown that the normalized initial rate is similar for both. However, it was noted that a more complex recovery of rice husk is required compared with the commercial ones.

Chicken eggshell is an abundant, safe, cheap waste material available from the poultry industry and has been used to immobilize β-galactosidase using crosslinking with glutaraldehyde [31]. The immobilized enzyme and free enzyme showed remarkable kinetic similarity, and the immobilized enzyme is stable and can be reused several times, making it a good catalyst to produce glucose from skim milk serum.

Lignocellulosic waste has been used to immobilize trypsin [32]. Corn cob powder (CCP) was used in the immobilization as a low-cost support with the goal of obtaining peptides with bioactive potential from cheese whey. The pre-treated support was activated with glyoxyl groups, glutaraldehyde and IDA-glyoxyl. The immobilization resulted in the retention of catalytic activity and resulted in a thermally stable enzyme at 65 °C, a value that was 1090-fold higher than that obtained with the free enzyme.

Biochar is a carbonaceous solid and recalcitrant material derived from the pyrolysis of waste biomass [33]. It has been engineered using various physical and chemical activation methods to alter its structural and physicochemical properties. Due to its low cost, presence of surface functional groups, porosity and moderate surface area, it has found use as a support material for the immobilization of enzymes including lipase, laccase and pepsin through adsorption and covalent attachment. 

### 2.5. Metal–Organic Frameworks

Metal–organic frameworks (MOFs) are composed of metal ions or clusters linked by organic ligands and are highly crystalline porous materials having high surface area, tunable ultra-high porosity, designable functionality and excellent thermal stability. Such a range of properties has made these attractive targets as supports for enzyme immobilization [34]. Extremely high surface area and tunable ultra-high porosity enable MOFs to achieve a very high loading of enzymes compared with traditional porous materials. Furthermore, it is feasible to tailor the pore aperture size and optimize it for a given enzyme. In doing so, the compact structure of an MOF enzyme can tightly confine the conformational structure of the encapsulated enzyme, leading to enzymes with extremely high stability. With such designable functionality and high thermostability, MOFs show much potential in the field of immobilized biocatalysis.

**Figure 3:**
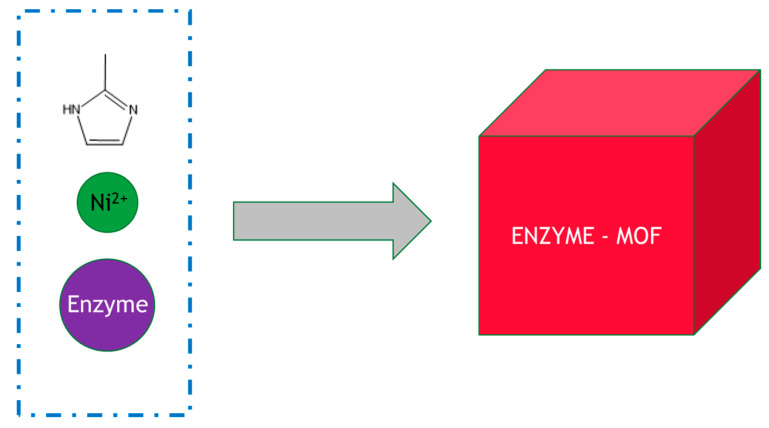
Synthesis of an enzyme–MOF composites in bulk solution. The reactants were added to the beaker and mixed with stirring.

The technology for MOF enzyme production is still at a very early stage, and the typical method of preparation is by simply mixing the enzymes, metal ions and organic ligands under ambient conditions in bulk solution, for example, glucose oxidase, zinc nitrate and 2-methylimidazole, as shown in Figure 3 [35]. This approach, whilst simple and straightforward to perform, is easy to scale up but generally requires improvement. For example, horseradish peroxidase, lipase and catalase embedded in zeolitic imidazolate MOF frameworks such as ZIF-8 and ZIF-90 only displayed less than ~10% activity of their free soluble counterparts. A marked increase in activity can be achieved by using microfluidic methods. The use of a three-way mixing scheme inside a microfluidic laminar flow system allows precisely controlled diffusive mixing conditions where one component can be added to the system with precise timing. In a surprising discovery, in flow mode, a gradient of concentration (of zinc nitrate and 2-methylimidazole) was generated that caused structural defects in the resulting MOF–enzyme composite. This in turn yielded an immobilized biocatalyst of much higher activity: instead of a typical 10% activity of the recovered MOF–enzyme, it had equivalent activity to the soluble enzyme, representing a significant step forward in this approach. The increased glucose oxidase activity was rationalized through enhanced substrate accessibility.

Whilst still embryonic, this approach for immobilizing enzymes holds much potential not only for biocatalysis but also in biosensing and nanomedicine.

### 2.6. Controlled Pore Glass 

**Figure 4:**
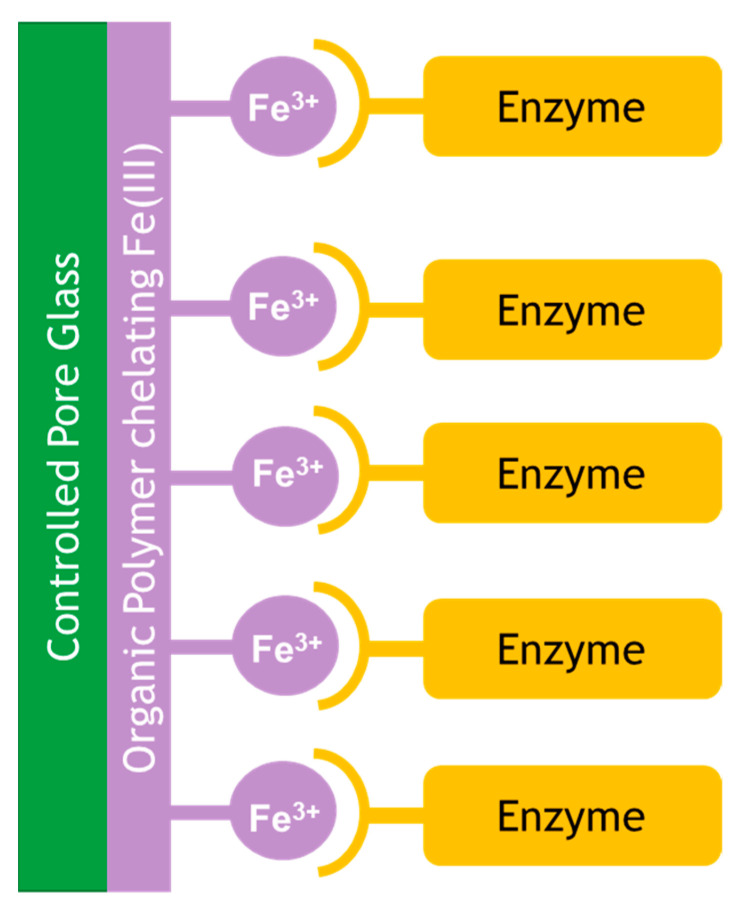
EziG binding of enzyme via Fe(III) coated to an organic polymer coated on controlled pore glass.

Controlled pore glass has been widely reported as a support for enzyme immobilization. EziG is a material based on controlled pore glass, which is coated with an organic polymer and chelated with Fe(III) for well-established His-tag binding (via an oligomeric his_6_-homopeptide) as shown in Figure 4 [36,37]. 

Because of efficient mass transfer through interconnecting pores and selective and non-destructive binding through His-tags, a high enzyme mass loading can be reached without the loss of activity caused by a high degree of diffusion limitation and deactivation. His-tag binding to Fe(III) was used rather than the commonly used Ni(II) or Co(II), as it gives a stronger (albeit less specific) bond, resulting in less or no leaching [38]. The authors show that *Candida antarctica* lipase B (CalB) can be efficiently expressed (6.2 g L^−1^) in *Escherichia coli* by utilizing an *N*-terminal tag cassette and the XylS/Pm expression system in a fed-batch bioreactor; subsequent direct binding to EziG from crude extracts resulted in an immobilized catalyst with superior activity to Novozym 435.

### 2.7. Magnetic Nanoparticles

Magnetic nanoparticles (MNP) have gained prominence in recent years as versatile carriers and supporting matrices for immobilization enzymes. Like MOFs described above, they have exceptional properties such as large surface area, large surface-to-volume ratio and high mass transfer capacity. More importantly, despite their nano-size (they may be just 10–20 nm particles), they can be easily separated and recovered by applying an external magnetic field. Several metals, oxides and alloys may be used as magnetic nanoparticles, but the most common type is iron oxide (Fe_3_O_4_) due to its high biocompatibility, non-toxicity and ease with which it may be used to bind enzymes. 

A wide variety of enzymes including oxidoreductases, hydrolases and transferases have been immobilized and insolubilized on the surface of functionalized MNPs to develop stable catalytic systems with easy separation and ability to recycle repeatedly. This subject was recently reviewed [39].

In the area of biocatalysis, examples of the application of magnetic nanoparticles include lipases immobilized for the epoxidation of oleic acid [40]. Highly stable and easily recyclable hybrid magnetic cross-linked lipase aggregates were prepared by co-aggregation of lipase aggregates with nonfunctionalized magnetic nanoparticles and subsequent chemical cross-linking with glutaraldehyde. The magnetic cross-linked lipase particles exhibited higher thermostability, storage stability and reusability than standard cross-linked enzyme aggregates. High conversion yield for the epoxidation of oleic acid using H_2_O_2_ as the oxidizing agent was achieved.

Another example relates to the use of nitrile hydratase for the production of nicotinamide from a nitrile [41]. A complex of silica, tannic acid and Fe_3_O_4_ particles was used as the basis for immobilizing nitrile hydratase, cross-linking the enzyme with glutaraldehyde. Its application for the hydrolysis of 3-cyanopyridine was demonstrated, giving significantly higher yields than for the use of the soluble non-immobilized enzyme. The recyclability of the immobilized enzyme was also shown with a simple magnetic recovery.

In a third example, epoxy-functionalized magnetic nanoparticles were prepared and used as a solid support for covalent immobilization and stabilization of benzoylformate decarboxylase from *Pseudomonas putida* [42]. This enzyme performs a carbon–carbon bond formation through a cross acyloin reaction of benzaldehyde with acetaldehyde to form (*S*)-2-hydroxypropiophenone. Immobilization was accomplished by a two-step mechanism, where firstly the enzyme was physically adsorbed onto the surface of the functionalized magnetic particles, then secondly enzyme surface nucleophiles (such as lysine) reacted with the epoxy groups. The covalently bound enzyme was characterized in terms of its activity and stability for the formation of (*S*)-2-hydroxypropiophenone. The activity of the immobilized enzyme was determined to be 53.0% related to the activity of the free enzyme. The immobilized biocatalyst retained 95% of its original activity after five reaction cycles.

**Figure 5:**
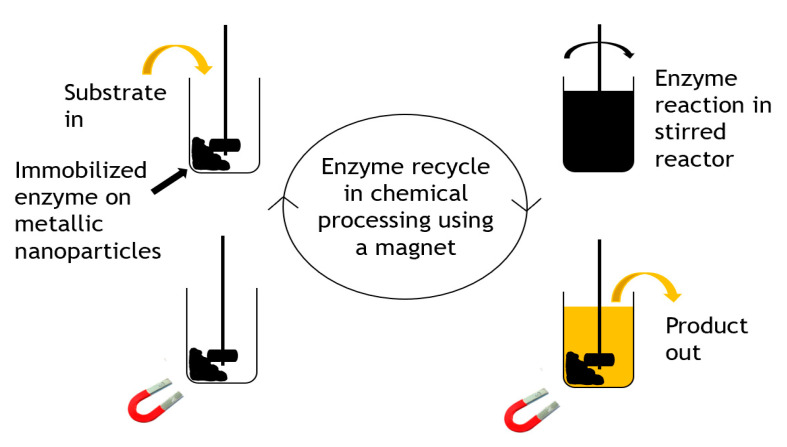
Recycling strategy for immobilized magnetic enzymes.

Carbon-coated metallic nanoparticles [43] were applied as useful supports for enzyme immobilization of three enzymes including β-glucosidase, α-chymotrypsin, and lipase B. They were used because of their large surface area, high magnetic saturation and manipulatable surface chemistry. The carbon-coated cobalt nanoparticles were chemically functionalized using diazonium chemistry, activated for bioconjugation with *N*,*N*-disuccinimidyl carbonate, and subsequently used in enzyme immobilization. The enzyme–particle conjugates formed retained their activity and stability after immobilization and were efficiently recycled from mL to L scales in short recycle times. The reaction was performed at a15 L scale and recycling was demonstrated, as shown in Figure 5.

Whilst no large-scale commercial applications are obvious yet, as an immobilization technology, the use of magnetic nanoparticles does show significant promise for companies with the technology base to manufacture and apply them. Certainly, aspirations for large-scale use are evident, for example, in reports for biodiesel and bioethanol. Niche applications in biocatalysis may emerge, for example, where heterogeneous mixtures of insoluble biocatalyst and product (or residual substrate) occur and filtration or centrifugation fails to selectively remove the biocatalyst for recovery. A magnetic approach could be a facile and selective way to recover an enzyme.

## 3. Integrating Immobilization into Developing Biocatalytic Technology

Biocatalytic technology is developing rapidly in numerous directions that go well beyond the simple concept of a batch reactor biotransformation comprising an immobilized enzyme and substrate. For example, four concepts are reported here to illustrate this, where immobilized enzymes are finding application in innovative new ways. 

### 3.1. Flow Biocatalysis

The first is in flow biocatalysis, a subset of the rapidly expanding field of flow chemistry. The use of immobilized enzymes in a packed column where a substrate flows through the biocatalyst is well established. Attention is now turning to the extension of this concept into general chemical flow processes and continuous synthesis, including microreactors, which may have multiple steps and where a spatial arrangement of multiple enzymes may be desirable. The use of enzyme immobilization for flow biocatalysis has been reviewed [44,45,46], and some examples follow. 

**Figure 6:**
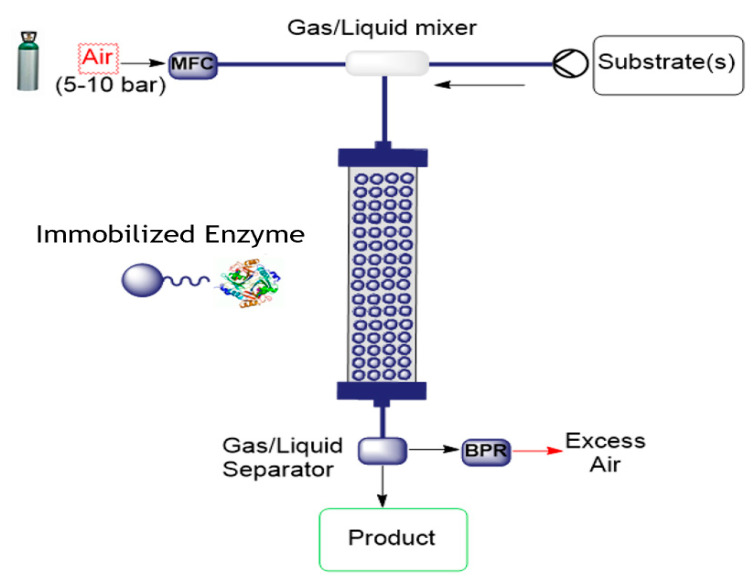
Flow set-up for air-mediated oxidation using immobilized enzymes (MFC = mass flow controller; BPR = back pressure regulator).

Oxidative O_2_-dependent biotransformations find many valuable potential applications for chemical synthesis, but their development to an efficiency required in fine chemicals manufacturing has proven challenging due to inefficiencies in delivering required amounts of oxygen. Thermodynamic and kinetic limitations of supplying O_2_ to the enzymatic reaction combine to limit conversion efficiency. Continuous flow microreactor technology provides one approach to solving this problem by expanding process conditions to a medium-pressure range, enabling biotransformations to be conducted in a single liquid phase at greatly elevated concentrations of dissolved O_2_. This was demonstrated for the two enzymes glucose oxidase and d-amino acid oxidase for the conversion of glucose to D-glucono-δ-lactone and d-methionine to the α-ketoacid, respectively [47]. These two biotransformations were demonstrated with a packed-bed reactor containing oxidase and catalase co-immobilized on porous beads to demonstrate catalyst recyclability and operational stability during continuous high-pressure conversion. Performance was impressive; product concentrations of up to 80 mM were obtained at low residence times (1–4 min), with up to 360 reactor cycles at constant product release and near-theoretical utilization of the O_2_ supplied. The set-up is illustrated in Figure 6.

As a second example, 2-*O*-(α-d-glucopyranosyl)-*sn*-glycerol is a natural osmolyte produced industrially for application as an active cosmetic ingredient. The biocatalytic process involves selective trans-glucosylation of glucose from sucrose to glycerol catalyzed by sucrose phosphorylase. This enzyme was immobilized onto the walls of a microchannel reactor where the walls were coated with aluminum oxide [48]. Continuous production of the product was shown over 16 days, equivalent to 916 batch reactor cycles, using 0.87 M sucrose and 2 M glycerol (86% conversion), demonstrating high volume efficiency and the basis of a continuous process.

The same enzyme was utilized for sugar phosphorylation reactions, where sucrose and inorganic phosphate yield α-d-glucose-1-phosphate [49]. Sucrose phosphorylase was immobilized on a microchannel surface and retained about 70% of soluble activity. High residual activity could still be retained after 690 reactor cycles, where synthesis of α-d-glucose-1-phosphate occurred with a productivity of ~14 mM min^−1^ at 50% substrate conversion (50 mM).

Flow biocatalysis with immobilized enzymes shows much potential in effecting synthetic transformations, and we may anticipate more examples and processes reaching commercial reality in the near future.

### 3.2. 3D-Printed Biocatalytic Scaffolds

**Figure 7:**
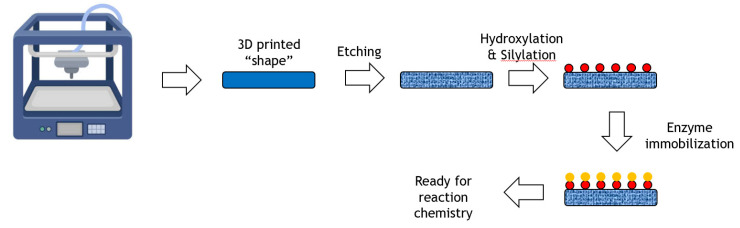
Immobilization of an enzyme onto a 3D printed shape.

The application of 3D-printing with its potential to achieve rapid prototyping of complex geometries is transforming the production of materials in industrial design, construction, tissue engineering and other fields. A generalized process from 3D-printer to immobilized enzyme is shown in Figure 7.

A novel enzyme immobilization strategy utilizing 3D-printing technology was reported where various types of scaffolds were first fabricated with a 3D-printed C-PLA (carbon fibre reinforced polylactic acid) matrix [50]. After chemical modification with a piranha solution, peracetic acid and a silane coupling agent, the resultant scaffolds achieved a high specific surface area with large numbers of active groups on the surface. Upon subsequent treatment with different silane coupling agents, penicillin G acylase (PGA), protease, glycosidase and lipase were successfully immobilized on the modified C-PLA scaffolds. The performance of these enzymes was assessed and found to have high synthetic potential. For example, the immobilized glycosidase was able to synthesize lactosucrose from sucrose and lactose at 20% weight/volume of each substrate, achieving around 150 g/L product. The system was successfully reused ten times with little loss of activity.

The synthesis of amoxicillin from 6-aminopenicillanic acid (6-APA) was also demonstrated using penicillin acylase. As with the example above, the enzyme was recovered and reused with little loss of activity.

We may anticipate further examples of enzyme immobilization on supports that have been 3D-printed where the printed structure is used as a cartridge in an integrated reactor. Alternatively, 3D printing may be used for developing microfluidic reactors where the scaffolds are incorporated into the reactor channels.

### 3.3. Multi-Enzymatic Cascade Reactions

There are numerous examples of products where more than one enzyme is utilized in its synthesis [51,52,53,54,55,56,57,58,59,60,61]. Whereas in the past such synthetic sequences may have been performed in separate steps in a linear manner, the use of cascade reactions is becoming more prevalent. In this approach several enzymatic steps are performed sequentially in a single reaction, thereby mimicking a metabolic pathway. As an alternative to the use of soluble enzymes, the use of immobilized enzymes in such artificial pathways is also a developing area and was recently reviewed [62]. 

In multienzyme cascades, several enzymes can work together with high efficiency. In such systems, each enzyme is assembled into a macromolecular structure that modulates reactivity by compartment formation or spatial organization. These defined multi-enzyme structures have a major advantage of substrate channeling. The active sites of the enzymes are close together, overcoming the diffusion limitations in the bulk solvent phase of the reaction by transferring intermediates from one active site to another, maintaining high local concentrations and therefore high enzyme activity.

Two main strategies have been followed where multiple enzymes are immobilized together. The first involves random co-immobilization and is arguably the simplest system. Generally, enzyme solutions are mixed with supports, and classical immobilization methods such as physical adsorption, covalent attachment or cross-linking are applied. It is also possible with such systems to co-immobilize co-factors such as NADH, PLP and FAD, which is of benefit to major biocatalyst classes such as keto reductases, ene reductases and transaminases [63]. 

**Figure 8:**
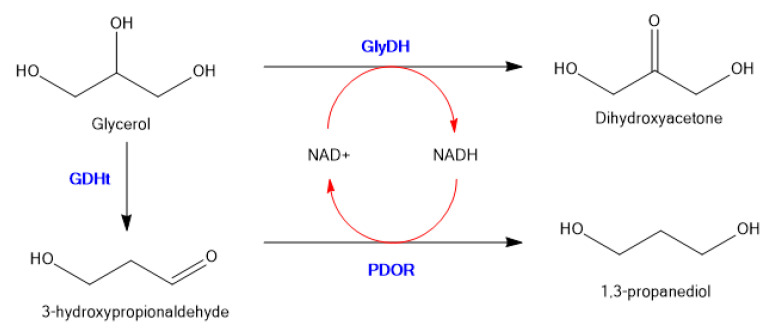
Three enzyme immobilized system to convert Glycerol to 1,3-propanediol with in situ co-factor recycling.

To illustrate random co-immobilization on an inorganic support, Wu et al. co-immobilized glycerol dehydrogenase (GDH), 1,3-propanediol oxidoreductase (PDOR) and glycerol dehydratase (GlyDH) on micro-flocculates of TiO_2_ nanoparticles, which were prepared by adsorption−flocculation with polyacrylamide [64]. The general scheme is shown in Figure 8.

This system converts glycerol to 1,3-propanediol in a single step with recycling of the nicotinamide co-factor. The stabilities of immobilized GDH against pH and temperature were significantly higher than those of free GDH, and simultaneous NAD(H) regeneration was feasible in the glycerol redox system, enabling the formation of 1,3-propanediol in yields of up to 11.6 g/L (46%). The porous and easily separable micro-flocculates of TiO_2_ nanoparticles with immobilized multienzymes were as efficient in terms of catalytic activity as the free enzymes.

An alternative approach to random co-immobilization is where the enzymes are immobilized onto themselves rather than onto a support in the form of cross-linked enzyme aggregates (combi-CLEAs) [65,66,67,68,69,70,71,72,73,74,75]. These can be prepared from crude enzyme extracts containing multiple enzymes, where enzymes are aggregated first with ammonium sulphate (a very rapid process) and then cross-linked by glutaraldehyde (a slower reaction) in a single-step process. An elegant example of this approach was reporting for a wine-making application with a combi-CLEA of α-l-arabino-furanosidase and β-d-gluco-pyranosidase [76]. To release the aroma content of wine, which is locked up in non-volatile disaccharide-terpene molecules, the α-l-arabino-furanosidase first releases a C5 sugar and then β-d-gluco-pyranosidase hydrolyses the resulting glucoside-terpene to release the volatile terpineol structures such as linalool, nerol and geraniol.

As discussed above, multienzyme immobilization via random co-immobilization may be the simplest strategy for improving overall catalytic activity and stability. This is not always possible if enzymes do not immobilize well in the required ratios or the functional groups on each enzyme do not all support the immobilization method. To overcome this and to mimic natural enzyme organization, immobilization by compartmentalization has been evaluated using various other approaches where enzymes are sequentially immobilized in shell structures. This is a relatively specialized approach and technically more demanding than combi-CLEAs or random co-immobilization on a single support.

### 3.4. Integrating Enzyme Immobilization and Protein Engineering

Until recently, enzyme immobilization and protein engineering have been treated very much as separate subjects. Generally, immobilization achieves reusable biocatalysts with improved operational stability and solvent resistance, whilst protein engineering aims to generate enzymes with increased performance under specific conditions by means of genetic manipulation, directed evolution and rational design. It has been proposed that these two concepts are now integrated together in a unified approach of immobilized biocatalyst engineering, where immobilization is addressed as an evolutionary pressure at the stage of enzyme screening during the protein engineering phase [77,78,79,80]. Whilst robust examples have yet to appear in the literature, we may anticipate such developments in the nearer future.

## 4. Conclusions

A review of recent literature in the field of enzyme immobilization shows a continuing and strong interest in this specialist area of biocatalysis. New types of supports and ways to immobilize enzymes are evident, for example, exploiting the use of sustainable low-cost waste as immobilization matrices. The integration of immobilization into newer areas of biocatalysis is evident too. A phalanx of examples includes the use of immobilized enzymes in flow chemistry, 3D-printed immobilized enzymes and immobilized multi-enzyme systems that catalyze artificial synthetic pathways that mimic metabolic processes.

The primary motivation for enzyme immobilization is to reduce biocatalyst cost contribution, and is expected to remain as the main driver, but as areas such as continuous manufacturing increase in prominence, then so too will the interest in integrating immobbbbilization technology into such processes increase. Enzyme catalysis is gaining great traction as success breeds success across many segments in chemical processing. Increased pressure on environmental control, cost reduction and higher quality requirements will see a cornucopia of enzyme processes emerging from conception to industrial realization as the technology of choice to meet these demands. Immobilization of these enzymes will stay in focus and, eventually, become a prerequisite.
